# Time trends and survival of marginal zone lymphoma over 25 years in Girona, Spain (1994–2018)

**DOI:** 10.1002/cam4.5935

**Published:** 2023-04-19

**Authors:** Carmen Auñón, Arantza Sanvisens, Estel Turon, Anna Vidal‐Vila, Montse Puigdemont, Gemma Osca‐Gelis, Arantxa Eraso, Rafael Marcos‐Gragera

**Affiliations:** ^1^ Radiation Oncology Department Institut Català d'Oncologia, Institut d'Investigació Biomèdica de Girona Dr. Josep Trueta (IDIBGI) Girona Spain; ^2^ Department of Medical Science Universitat de Girona Girona Spain; ^3^ Epidemiology Unit and Girona Cancer Registry Institut Català d'Oncologia, Pla Director d'Oncologia, Institut d'Investigació Biomèdica de Girona Dr. Josep Trueta (IDIBGI) Girona Spain; ^4^ Institut de Recerca Contra la Leucèmia Josep Carreras Girona Spain; ^5^ Registre de Tumors Hospitalari (RTH ICO‐ICS) Institut Català d'Oncologia, Hospital Universitari Dr. Josep Trueta, Institut d'Investigació Biomèdica de Girona Dr. Josep Trueta (IDIBGI) Girona Spain; ^6^ Department of Economy, Research Group on Statistics, Econometrics and Health (GRECS) Universitat de Girona Girona Spain; ^7^ CIBER of Epidemiology and Public Health (CIBERESP) Girona Spain; ^8^ Department of Nursing Universitat de Girona Girona Spain

**Keywords:** cancer, epidemiology, hematology, marginal zone lymphoma, population‐based

## Abstract

**Objective:**

To analyze the incidence, incidence trends, and survival of marginal zone lymphomas (MZLs) in Girona and to describe these indicators based on the location in the case of extranodal MZLs.

**Methods:**

Population‐based study of MZL collected in the Girona Cancer Registry, 1994–2018. Sociodemographic data, tumor location, and stage were obtained from clinical records. Crude (CR) and age‐adjusted (ASR_E_) incidence rates expressed per 100,000 person‐years (p‐y) were calculated. Joinpoint regression models were used for the trend analysis according to the MZL group. Five‐year observed and net survival were analyzed.

**Results:**

A total of 472 MZLs were included, 44 (9.3%) were nodal, 288 (61.0%) extranodal, 122 (25.9%) splenic, and the rest (*n* = 18) MZL, NOS. The CR for the MZL was 2.89 × 100,000 p‐y (95% CI: 2.63–3.15), the ASR_E_ was 3.26 × 100,000 p‐y (95% CI: 2.97–3.57), and the annual percentage change (APC) was 1.6 (95% CI: 0.5–2.7). The ASR_E_ for nodal MZL was 0.30 × 100,000 p‐y (95% CI: 0.22–0.41) and showed an APC of 2.9% (95% CI: −16.4–26.6). For extranodal MZL, the ASR_E_ was 1.98 × 100,000 p‐y (95% CI: 1.76–2.23) and the APC was −0.4 (95% CI: −2.0–1.2). The most frequent locations of this type of MZL were the gastric (35.4%), skin (13.2%), and respiratory system (11.8%). The ASR_E_ of the splenic MZL was 0.85 (95% CI: 0.71–1.02) with an APC of 12.8 (95% CI: 2.5–24.0). The 5‐year net survival of MZL was 82.1% (95% CI: 76.3–86.5).

**Conclusions:**

This study reveals differences in the incidence and trend of the incidence of MZL according to the subgroup, showing a significant increase in the overall MZL mainly due to splenic MZL type.

## 1 INTRODUCTION

Marginal zone lymphomas (MZLs) are indolent type B non‐Hodgkin lymphoid neoplasms with a relatively good prognosis.^1^ These types of neoplasms represent around 3% of all lymphoid neoplasms.^2^, ^3^

According to the World Health Organization (WHO) classification of hematopoietic and lymphoid tissue tumors, MZLs are classified into three large groups: splenic marginal zone lymphoma (SMZL), nodal marginal zone lymphoma (NMZL), and extranodal MZL, otherwise known as MALT (mucosa‐associated lymphoid tissue) lymphomas.^4^, ^5^ The incidence of MZL varies between 0.5 and 2.92 cases per 100,000 person‐years depending on the geographical area, with MALT lymphomas being the most incident. The median age at diagnosis is around 70 years and the male‐to‐female ratio ranges from 0.9 to 1.78, according to different studies.^2^, ^6^, ^7^, ^8^, ^9^, ^10^, ^11^, ^12^, ^13^, ^14^ Furthermore, an increase in incidence has been observed in recent years both in overall MZLs and in the different subgroups.^2^, ^3^, ^6^, ^11^, ^12^ It is estimated that the observed 5‐year survival is > 70% and the net survival is between 76% and 91%, depending on the region.^1^, ^6^, ^7^, ^8^, ^12^, ^15^, ^16^

The etiologic factors of MZL differ according to subgroup; in addition, in the case of MALT lymphomas, they also vary with the anatomical location of the tumor. Specifically, MALT‐type MZLs are associated with a persistent immune response and have therefore been associated with autoimmune diseases or microbial infections, such as Sjögren's syndrome when localized in the salivary glands, Hashimoto's thyroiditis in thyroid lymphoma, *Helicobacter pylori* (HP) gastritis in gastric lymphoma, *Chlamydia psittaci* in lymphomas located in the ocular adnexa, *Campylobacter jejuni* in intestinal lymphoma, or *Borrelia burgdorferi* in the skin.^17^, ^18^, ^19^, ^20^, ^21^

Hepatitis C virus (HCV) has been described as a risk factor for nodal and splenic MZL, although it has also been related to MALT lymphomas of the salivary glands, skin and ocular adnexa.^21^, ^22^, ^23^ In fact, both in the era of HCV treatment with interferon and currently with the new direct‐acting antivirals, a regression of lymphomas has been observed together with a sustained viral response.^24^, ^25^ Localized MZLs are mainly represented by extranodal MZLs, although around a quarter of MALT lymphomas are disseminated at the time of diagnosis.^26^, ^27^, ^28^

Population‐based epidemiological data examining the different MZLs types are scarce, and most have focused on one of the three large groups described above^6^, ^7^, ^8^, ^29^, ^30^. A study published in 2014 with data from the *U.S. Surveillance, Epidemiology and End Results (SEER) Program population‐based registries* described the most common extranodal locations as being the stomach, with an incidence of 3.8 per one million person‐years, followed by the spleen (1.6) and the eye/adnexa (1.4).^3^

The objective of this study is to analyze the incidence, incidence trend and survival of MZL in the province of Girona over a 25‐year period according to the anatomical location of MALT‐type lymphomas.

## 2 METHODS

MZL cases were obtained from the Girona population‐based Cancer Registry (GCR), Spain, collected during the period 1994–2018. The GCR covers the population of Girona province, located in northeastern Spain, with 749,656 inhabitants in 2018 according to the National Statistics Institute. The GCR is a member of the International Agency for Cancer Registries (IACR). Procedures for cancer registries and coding rules were used in accordance with the standards of the International Agency for Research on Cancer (IARC) standards.^31^

For this study, the cases were morphologically coded according to the International Classification of Diseases for Oncology, 3rd Revision (ICD‐O‐3)^32^ and classified into four groups based on location and morphology, following the WHO Classification of Tumors of Hematopoietic and Lymphoid Tissues^4^, ^5^ and the ICD‐O‐3 code. Specifically: (1) SMZL (M‐9689 with topographic code C42.2); (2) NMZL (M‐9699 with topographic code C77.0‐C77.9); (3) extranodal MZL (M‐9764 and M‐9699 excluding topographic codes C77.0‐C77.9, C42.0, C42.1); and (4) MZL NOS, (M‐9699 and topographic code C42.0 or C42.1).

In turn, extranodal MZLs were classified into seventeen subgroups: (3.1) gastric digestive (C16—stomach); (3.2) nongastric digestive (C17—small intestine, C18—colon, C20—rectum, C26—other digestive organs); (3.3) skin (C44); (3.4) salivary glands (C07—parotid gland, C08—other glands); (3.5) neck, oral cavity and pharynx (C05—palate, C06—other parts of the mouth, C09—tonsil, C10—oropharynx, C11—nasopharynx, C14—other sites in lip, oral cavity and pharynx); (3.6) central nervous system (C70—meninges, C72—spinal cord, cranial nerves and other parts of central nervous system); (3.7) liver and intrahepatic bile ducts (C22); (3.8) nasal cavity and middle ear (C30); (3.9) respiratory system (C34—bronchus and lung, C39—other intrathoracic sites and organs); (3.10) thymus (C37); (3.11) thyroid gland (C73); (3.12) connective, subcutaneous and other soft tissue (C49); (3.13) breast (C50); (3.14) uterus, NOS (C55); (3.15) testis (C62); (3.16) urinary bladder (C67); (3.17) eye and adnexa (C69).

All cases were retrospectively reviewed to collect information on the location and extent of the disease, as well as pathologic history and comorbidity at the time of MZL diagnosis such as history of autoimmune and/or inflammatory diseases, history of infection with hepatitis B virus (HBV), HCV, HP and history of pulmonary tuberculosis (TBC).

For patients with SMZL, the level of risk was obtained using the simplified risk stratification system described by Montalban et al.^33^ Patients' vital status was obtained by crossing data with the Mortality Registry of Catalonia, National Death Index and review of medical records up to December 31, 2019.

### 2.1 Statistical analysis

Descriptive statistics for quantitative variables were expressed as the median and interquartile range (IQR), and for qualitative variables as absolute frequencies and percentages. To analyze differences in the variables of interest between study groups, the Mann–Whitney U test or Chi‐squared test was used, depending on the data distribution.

Crude incidence rates (CR) and age‐adjusted incidence rates based on the 2013‐European standard population (ASR_E_) and world standard population (ASR_W_) were calculated and expressed per 100,000 person‐years (p‐y). Rates were calculated based on sex, age, MZL subgroup and anatomic location for extranodal MZL. Joinpoint regression models were used for trend analysis, estimating trend change points and annual percentage change (APC). Trend analysis was performed for the overall MZL and the main subgroups.

The Kaplan–Meier method was used to calculate the 5‐year observed survival and the log‐rank test to establish differences in survival according to the MZL subgroup. Net survival was calculated using the Pohar‐Perme method. No cases diagnosed only by death or autopsy certificate were identified.

Data analysis was performed using R software (version 4.0.3), Joinpoint Regression Program version 4.9.0.0 (Statistical Methodology and Applications Branch, Surveillance Research Program, National Cancer Institute), and Stata (version 14.2, StataCorp LLC). Differences were considered statistically significant at *p* < 0.05.

### 2.2 Ethics statements

The participating cancer registry has data management policies in place to preserve patient confidentiality, including ethical approval from local obligatory bodies. The study was carried out in accordance with the guidelines of the Declaration of Helsinki and reviewed by the institutional review board at the Dr. Josep Trueta Hospital Universitari de Girona (approval number 2022.063). It did not require informed consent in line with laws 14/1986 and 33/2011, on Spanish general and public health, as well as law 8/2001 of June 14, regarding the 2001–2004 Statistical Plan for Catalonia, which recognizes the GCR as a statistical database.

## 3 RESULTS

A total of 472 patients diagnosed with MZL in the province of Girona between 1994 and 2018 were included: 44 (9.3%) NMZL; 288 (61.0%) extranodal MZL; 122 (25.9%) SMZL; and the remainder, 18 (3.8%) LZM, NOS.

Overall, the median age at diagnosis was 68 years [IQR: 57–77], and 51.5% were diagnosed in men. No statistically significant differences were observed in the age and sex of the patients according to the MZL subgroup.

**FIGURE 1:**
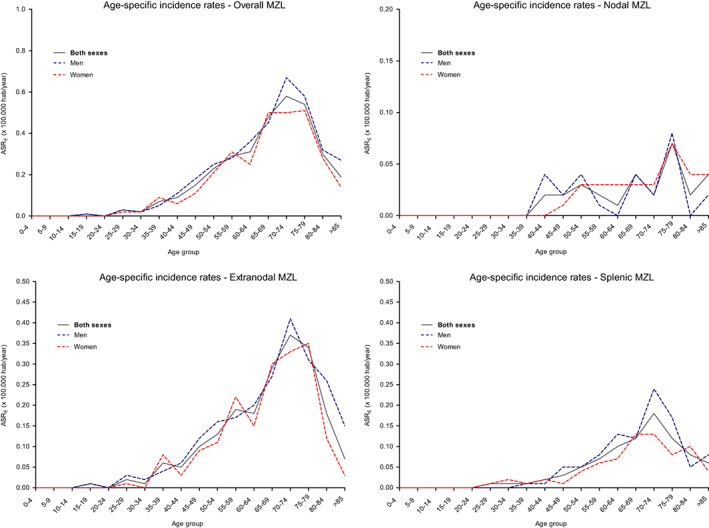
Age‐specific incidence rates of overall and subtype of MZL in Girona, 1994–2018

The CR for all MZLs was 2.89 × 100,000 p‐y (95% confidence interval [CI]: 2.63–3.15), and the ASR_E_ was 3.26 × 100,000 p‐y (95% CI: 2.97–3.57). Table 1 shows the CR and ASR_E_ for the different MZL subgroups according to sex. Incidence rates were observed to increase with age, both for overall MZL and for the different subgroups; Figure 1 shows the specific rates by age.

**TABLE 1:** Distribution, crude, and age‐adjusted incidence rates of MZL in Girona, 1994–2018 (European standard population)

	Total *n* (%)	Median age [IQR]	CR (95% CI)	ASR_E_ (95% CI)	Men ASR_E_ (95% CI)	Women ASR_E_ (95% CI)	Ratio M/W
MZL—Overall	472 (100)	68.0 [57.0, 77.0]	2.89 (2.63–3.15)	3.26 (2.97–3.57)	3.58 (3.14–4.08)	3.00 (2.62–3.42)	1.19
1. SMZL	122 (25.9)	69.5 [61.0, 76.0]	0.75 (0.61–0.88)	0.85 (0.71–1.02)	0.99 (0.76–1.27)	0.73 (0.55–0.95)	1.36
2. NMZL	44 (9.3)	70.5 [53.5, 77.0]	0.27 (0.19–0.35)	0.30 (0.22–0.41)	0.28 (0.17–0.45)	0.30 (0.19–0.46)	0.93
3. Extranodal MZL—MALT	288 (61.0)	68.0 [56.0, 76.0]	1.76 (1.56–1.96)	1.98 (1.76–2.23)	2.20 (1.86–2.60)	1.82 (1.53–2.16)	1.21
3.1 Digestive—gastric	102 (21.6)	66.0 [53.0, 74.0]	0.62 (0.50–0.74)	0.70 (0.57–0.85)	0.85 (0.65–1.11)	0.57 (0.41–0.77)	1.49
3.2 Digestive—no gastric	18 (3.8)	71.5 [65.0, 79.0]	0.11 (0.06–0.16)	0.12 (0.07–0.20)	0.13 (0.06–0.26)	0.12 (0.05–0.22)	1.08
3.3 Skin	38 (8.1)	61.5 [56.0, 72.0]	0.23 (0.16–0.31)	0.26 (0.18–0.36)	0.27 (0.16–0.43)	0.26 (0.15–0.41)	1.04
3.4 Salivary glands	27 (5.7)	67.0 [57.0, 74.0]	0.17 (0.10–0.23)	0.19 (0.12–0.28)	0.13 (0.06–0.26)	0.25 (0.14–0.39)	0.52
3.5 Oral cavity and pharynx	18 (3.8)	62.0 [47.0, 71.0]	0.11 (0.06–0.16)	0.12 (0.07–0.19)	0.18 (0.09–0.32)	0.07 (0.02–0.16)	2.57
3.6 Central nervous system	2 (0.4)	57.0 [54.0, 60.0]	0.01 (0.00–0.03)	0.01 (0.00–0.05)	0.013 (0.00–0.10)	0.015 (0.00–0.08)	0.87
3.7 Liver	1 (0.2)	68.0 [68.0, 68.0]	0.006 (0.00–0.02)	0.007 (0.00–0.04)	0.015 (0.00–0.10)	0 (NA‐0.05)	—
3.8 Nasal and ear	1 (0.2)	82.0 [82.0, 82.0]	0.006 (0.00–0.02)	0.006 (0.00–0.04)	0.015 (0.00–0.10)	0 (NA‐0.05)	—
3.9 Respiratory system	34 (7.2)	72.0 [67.0, 78.0]	0.21 (0.14–0.28)	0.24 (0.17–0.34)	0.29 (0.17–0.47)	0.21 (0.12–0.35)	1.38
3.10 Thymus	1 (0.2)	43.0 [43.0, 43.0]	0.006 (0.00–0.02)	0.005 (0.00–0.04)	0.01 (0.00–0.09)	0 (NA‐0.05)	—
3.11 Thyroid	5 (1.1)	72.0 [51.0, 79.0]	0.031 (0.00–0.06)	0.034 (0.01–0.08)	0.013 (0.00–0.10)	0.05 (0.01–0.13)	0.26
3.12 Connective tissues	2 (0.4)	75.5 [71.0, 80.0]	0.012 (0.00–0.03)	0.013 (0.00–0.05)	0.016 (0.00–0.10)	0.01 (0.00–0.06)	1.6
3.13 Breast	7 (1.5)	68.0 [64.0, 78.0]	0.04 (0.01–0.07)	0.05 (0.02–0.10)	0 (NA‐0.08)	0.09 (0.04–0.20)	0
3.14 Uterus	1 (0.2)	77.0 [77.0, 77.0]	0.012 (0.00–0.04)	0.013 (0.00–0.08)	—	0.01 (0.00–0.08)	—
3.15 Testis	1 (0.2)	88.0 [88.0, 88.0]	0.01 (0.00–0.04)	0.02 (0.00–0.12)	0.02 (0.00–0.12)	—	—
3.16 Bladder	1 (0.2)	73.0 [73.0, 73.0]	0.006 (0.00–0.02)	0.007 (0.00–0.04)	0.016 (0.00–0.10)	0 (NA‐0.05)	—
3.17 Eye and annexa	29 (6.1)	69.0 [51.0, 78.0]	0.18 (0.11–0.24)	0.19 (0.13–0.28)	0.23 (0.13–0.39)	0.16 (0.09–0.28)	1.44
4. MZL, NOS	18 (3.8)	71.5 [64.0, 81.0]	0.11 (0.06–0.16)	0.13 (0.07–0.20)	0.11 (0.04–0.24)	0.14 (0.07–0.25)	0.79

**FIGURE 2:**
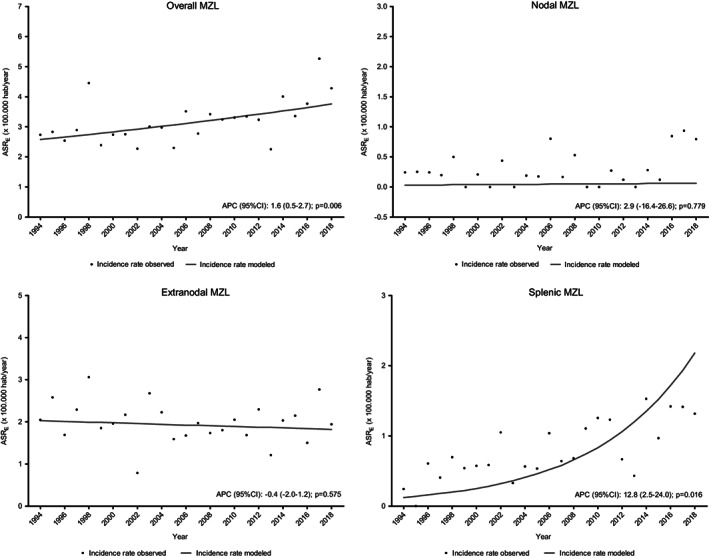
Trends of MZL incidence rates in Girona 1994–2018

The trend for the incidence of MZL was increasing and statistically significant, with an APC (95% CI): 1.6 (0.5–2.7), *p* = 0.006, with no points of trend change observed. The incidence trend analyses are shown in Figure 2.

Table S1 shows age‐adjusted rates for the world population, by sex.

### 3.1 Splenic MZLs

Of the 122 cases of splenic MZL, 55.7% were diagnosed in males. The median age was 69.5 years [IQR: 61–76]; 64.0% of cases were diagnosed by means of histology of the primary tumor and 34.4% by means of cytology. For the 48 cases in which the level of risk could be determined, 56.2% (27/48) were medium risk and 39.6% were low risk.

The CR for splenic MZLs was 0.75 × 100,000 p‐y (0.61–0.88) and the ASR_E_ was 0.85 × 100,000 p‐y (0.71–1.02), with a male/female ratio of 1.36. The age‐specific rates were similar to those of the other subgroups (Figure 1); however, the APC was statistically significant (12.8%, 95% CI: 2.5–24.0, *p* = 0.016) (Figure 2). Among those patients who had serology for HCV and HBV, 6.7% (3/45) and 17.0% (8/47) were positive, respectively.

### 3.2 Nodal LZMs

The median age of the 44 patients diagnosed with nodal MZL was 70.5 years [IQR: 53.5–77.0], it being older in women than in men (75.5 years [IQR: 62.0–83.5] and 60 years [48–76], respectively; *p* = 0.015); 95.4% were diagnosed by primary tumor histology and the majority (68.9%, 20/29 cases) were diagnosed at advanced stages (III and IV). A total of 14 patients had serology for HCV and HBV, which were positive in 28.6% and 14.3%, respectively.

The CR incidence for nodal MZLs was 0.27 × 100,000 p‐y (95% CI: 0.19–0.35), and the ASR_E_ was 0.30 × 100,000 p‐y (95% CI: 0.22–0.41), with a male/female ratio of 0.93 (Table 1). The incidence trend remained stable over time (Figure 2).

### 3.3 Extranodal MZLs or MALT‐type lymphomas

The median age of patients diagnosed with extranodal MZL was 68 years [IQR: 56–76]; 97.2% were diagnosed by primary tumor histology and the majority of cases in which staging was obtained (76.9%, 150/195 cases) were diagnosed at localized stages (I and II).

For extranodal MZLs, the CR and ASR_E_ were 1.76 × 100,000 p‐y (95% CI: 1.56–1.96) and 1.98 × 100,000 p‐y (95% CI: 1.76–2.23), respectively. The overall APC was −0.4 (95% CI: −2.0–1.2), *p* = 0.575. In this subgroup of MZLs, the most frequent locations were gastric (35.4%), skin (13.2%), respiratory system (11.8%), eyes and adnexa (10.1%), salivary glands (9.35%), nongastric digestive system (6.2%), and oral cavity and pharynx (6.2%).

**FIGURE 3:**
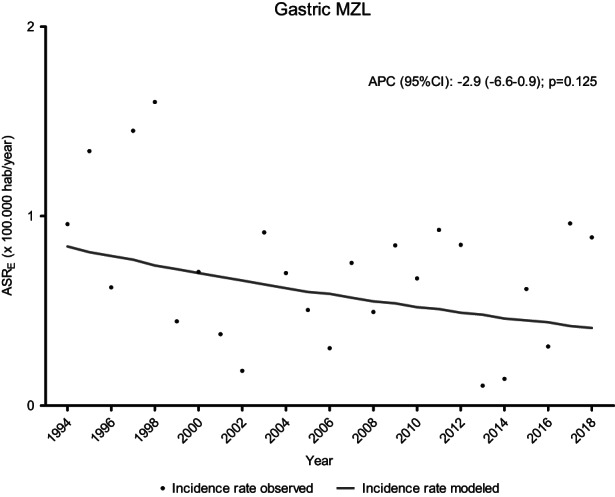
Trends of incidence rates in gastric extranodal MZL, Girona 1994–2018

In the specific case of gastric extranodal MZLs for which HP determination was obtained, 78% (46/59) were positive. Figure 3 shows the incidence trend for extranodal MZLs of gastric origin. Among nongastric digestive MZL tumors, 11% (2 cases) had celiac disease.

Regarding MZLs located in the skin, it should be noted that one in two (20/38 cases, 52.6%) were located in the head, neck, or upper extremities.

In cases located in the respiratory system, it was observed that four (11.7%) had a history of pulmonary TBC and one had pulmonary fibrosis.

Finally, it should be noted that 14.8% of the patients with tumors located in the salivary glands had a history of Sjögren's syndrome (11% in males and 17% in females). These salivary gland tumors, as well as those of the thyroid gland, were more frequent among women (Table 1); by contrast, the male/female ratio of 2.57 for those located in the oral cavity and pharynx stands out.

### 3.4 Survival

A total of 209 patients (44.3%) died during follow‐up. The median follow‐up was 5.7 years [IQR: 2.3–10.6], with a cumulative follow‐up of 3388 p‐y. The median survival was 12 years, and the observed 5‐year survival was 73.7% (95% CI: 69.2–77.7). No statistically significant differences were observed by the MZL subgroup, with survival being 67.4%, 77.3%, and 66.2% for splenic, extranodal, and nodal MZL, respectively (*p* = 0.526).

**FIGURE 4:**
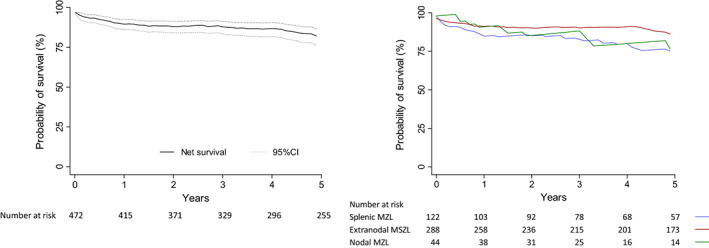
Five‐year net survival of overall MZL and according to subgroup

The 5‐year net survival for MZL was 82.1% (95% CI: 76.3–86.5), with 74.7% (95% CI: 62.4–83.4) for splenic, 85.6% (95% CI: 78.2–90.6) for extranodal and 76.4% (95% CI: 48.2–90.5) for nodal. Figure 4 shows the 5‐year net survival of patients with MZL overall and by subgroup.

## 4 DISCUSSION

Epidemiologic data on hematologic malignancies are scarce. To the best of our knowledge, this is the first population‐based study from southern Europe that shows contemporary and specific epidemiologic data for MZL based on anatomical location; the SEER program in the United States has also published population epidemiologic indicators of MZL based on site.^3^

The incidence rate observed in this study conducted between 1994 and 2018, which was 3.26 per 100,000 inhabitants/year (ASR_E_) and 1.59 per 100,000 inhabitants/year (ASR_W_), was higher than that of other comparable population studies.^8^, ^11^, ^34^, ^35^ In the United Kingdom, the standardized incidence rates for MZLs were 2.3 for the period 2004–2009 and 2.6 for the period 2004–2012,^8^, ^34^ while data recently published from Spain show a rate of 1.84 for the period 2002–2013.^35^ These differences could be due to different reasons, among which it is worth highlighting a greater knowledge of this pathology and recognition by pathologists, as well as improvements in diagnostic methods (morphology, immunophenotype and molecular biology) and coding.^2^, ^6^

Overall, the MZL incidence was found to be similar in men and women and increased with age. In our study, the median age was 68 years and the male/female ratio was 1.19, data similar to those already published by other series.^3^, ^6^, ^7^, ^8^, ^9^, ^10^, ^11^, ^12^ However, extranodal MZL in certain locations showed different incidence rates according to sex, as we will highlight later on.

As observed elsewhere, the most incident subgroup in this study was extranodal MZL.^3^, ^8^, ^11^, ^12^, ^13^, ^14^ Depending on the location, the highest incidence rates were observed in gastric digestive tumors, skin, respiratory system, eyes and adnexa, and salivary glands, similar to SEER data published in 2014.^3^

Extranodal MZLs of the digestive tract were the most frequent, although we were able to observe a decreasing trend in their incidence. These lymphomas are closely related to HP infection,^20^, ^36^, ^37^ and the increase in the diagnosis and treatment of this infection has reduced the risk of this type of lymphoma. In our study, four out of five cases (78%) of gastric extranodal MZL showed this infection, similar to previous findings^38^, ^39^; in fact, its eradication is considered the main and most important step in the therapeutic strategy although certain cases with the genetic abnormality BIRC3::MALT1 do not respond to it.^39^, ^40^

MZL of the respiratory system, oral cavity and pharynx, eyes and adnexa were also found to be more common in men. Although there are not many population data to contrast with this result, both the study by Khalil et al.,^3^ and some case series on non‐Hodgkin's lymphomas of the respiratory system and oropharynx show data consistent with ours.^3^, ^41^, ^42^

In relation to cutaneous MZLs, it should be noted that, as with the SEER 3 data that have been published,^3^ the sublocations with the highest incidence were those with exposure to sunlight, such as the face, scalp and upper extremities, which may be related to ultraviolet radiation, as occurs with melanoma.^43^

Salivary and thyroid glands are the two MZL locations that were clearly more common in women than in men in our study, consistent with the results published using SEER3 data.^3^ The main risk factors described for extranodal MZLs of the salivary and thyroid glands were Sjögren's syndrome and Hashimoto's thyroiditis, respectively; in fact, both of these diseases that are of autoimmune origin are more frequent among women.^44^, ^45^ In our study, 17% of the cases of salivary gland MALT in women had a history of Sjögren's syndrome compared with 11% in men, and all cases of Hashimoto's thyroiditis were observed in women (25% vs. 0%).

As mentioned previously, an increase in MZL incidence has been observed over the years, although it is interesting to note that this trend has been at the expense of SMZLs. This increase in the incidence of SMZLs could be attributed to an improvement in diagnosis. Specifically, the description of the first histologic pattern attributable to SMZLs was made in 1992, and it is therefore plausible that it has gradually improved since then.^46^ In fact, the definitive diagnosis of SMZLs is based on the histology of the spleen, which is not always available. Without a histological result for the spleen, the differential diagnosis with other lymphoproliferative disorders would be compromised, although immunophenotyping and cytogenetics, which are increasingly more widespread, are of great help in this regard.^47^ On the other hand, persistent HCV infection, which is one of the etiological factors of this pathology due to chronic antigenic stimulation, could also have contributed to this growing trend in the incidence of this type of lymphoma over the years.

With regard to survival, we have observed that MZLs generally have a good prognosis, with 82% survival at 5 years, which has improved over the time (data not shown), a figure similar to those already published in other studies and the SEER database.^6^, ^7^, ^8^, ^12^, ^15^, ^16^ However, few studies have provided survival data by the MZL subgroup^16^; in fact, no statistically significant differences were observed in 5‐year survival according to the different subgroups in our study, with the survival of MALT‐type lymphoma being the highest, at 86%. This could, in part, be due to the eradication of the causal agent (HP) as first‐line treatment in gastric MALT cases attributable to this infection, which in turn are the most incident in this group. In addition, a significant proportion was diagnosed at an early stage, and these cases were considered indolent, with low rates of proliferation and low risk of progression.^39^

Regarding the 5‐year survival of nodal MZL and SMZL, it is estimated at around 75% in our study. In the case of SMZL, the majority of patients were observed to present an indolent course of the disease, although around 30% showed a worse prognosis and some underwent transformation to diffuse large B‐cell lymphoma.^48^, ^49^

The same was true for nodal MZL, with around 15% transforming into diffuse large B‐cell lymphomas, resulting in a less favorable prognosis.^50^, ^51^ Even though nodal MZL has been associated with chronic inflammation and infection, it was not possible to establish an association with the available data. In this regard, one of the main limitations that should be mentioned of this study was the lack of clinical data that could not be collected completely and exhaustively when reviewing the medical records, either to establish etiologic associations or the stage of the disease at the time of diagnosis This limitation is inherent to the retrospective nature of this study and the long period analyzed and, in turn, is an impairment to extrapolate the data to other populations given that they could have a different prevalence of the possible etiological factors of MZLs. In addition, the lack of a control group makes it difficult to establish associations with possible risk factors. On the other hand, the low number of cases in certain subgroups such as nodal MZLs may have limited the statistical power in the incidence trend analysis. In fact, the increasing trend that has been observed in the incidence of this type of lymphoma could not be established in this study.^3^ Finally, the lack of information on treatment regimens such as rituximab or other novel agents may difficult the interpretation of the survival results. However, the main strength of this work is based on providing contemporary and specific population data on MZLs according to anatomical location.

In conclusion, then, this study shows a general increasing trend in the incidence of MZLs. However, marked differences were observed according to subgroup and location. Changes in the diagnosis and treatment of certain etiologic factors such as HP infection may have influenced the epidemiologic indicators of MALT‐type lymphomas, while new direct‐acting antiviral treatments against HCV may play a key role in the incidence of SMZLs in the near future. In any case, MZLs showed a relatively good prognosis overall, regardless of the subgroup diagnosed.

## AUTHOR CONTRIBUTIONS

**Carme Auñón:** Conceptualization (equal); data curation (equal); investigation (equal); writing – original draft (equal). **Arantza Sanvisens:** Conceptualization (equal); formal analysis (equal); investigation (equal); writing – original draft (equal); writing – review and editing (equal). **Estel Turon:** Formal analysis (equal). **Anna Vidal‐Vila:** Data curation (equal). **Montse Puigdemont:** Data curation (equal). **Gemma Osca‐Gelis:** Data curation (equal). **Arantxa Eraso:** Supervision (equal). **Rafael Marcos:** Conceptualization (equal); supervision (equal).

## FUNDING INFORMATION

This work was partially funded by the Josep Carreras Leukemia Research Institute (grant number: FIJC1100) and the Agency for Management of University and Research Grants, Government of Catalonia (grant number: 2021 SGR 01511).

## CONFLICT OF INTEREST STATEMENT

None declared.

## Supporting information


Table S1.

## Data Availability

The dataset analyzed during the current study is not publicly available due to national regulations of cancer registry data. However, it is available from the corresponding author upon reasonable request.
