# Supplementary material for: Time trends and survival of marginal zone lymphoma over 25 years in Girona, Spain (1994–2018)
Source: Cancer Med. 2023 Apr 19;12(11):12343–53. doi: 10.1002/cam4.5935 (PMC10278498; doi:10.1002/cam4.5935)
Supplement: Supplementary file 1 — Table S1. [file CAM4-12-12343-s001.docx]

Supplementary Table 1. Age-adjusted incidence rates of MZL in Girona, 1994-2018 (World standard population).

|  | **ASR_W_ (95% CI)** | **Men**  **ASR_W_ (95%CI)** | **Women**  **ASR_W_ (95%CI)** |
| --- | --- | --- | --- |
| **MZL – Overall** | 1.59 (1.44-1.77) | 1.74 (1.52-2.01) | 1.46 (1.25-1.71) |
| **1.SMZL** | 0.41 (0.33-0.51) | 0.46 (0.35-0.62) | 0.36 (0.26-0.51) |
| **2.NMZL** | 0.14 (0.10-0.21) | 0.15 (0.09-0.27) | 0.13 (0.07-0.25) |
| **3. Extranodal MZL - MALT** | 0.99 (0.87-1.14) | 1.08 (0.91-1.31) | 0.91 (0.75-1.12) |
| 3.1 Digestive – gastric | 0.37 (0.29-0.46) | 0.44 (0.33-0.61) | 0.29 (0.21-0.44) |
| 3.2 Digestive – no gastric | 0.05 (0.03-0.11) | 0.06 (0.02-0.16) | 0.05 (0.02-0.16) |
| 3.3 Skin | 0.14 (0.10-0.22) | 0.15 (0.09-0.28) | 0.14 (0.08-0.26) |
| 3.4 Salivary glands | 0.09 (0.06-0.16) | 0.06 (0.03-0.17) | 0.12 (0.07-0.24) |
| 3.5 Oral cavity and pharynx | 0.07 (0.04-0.13) | 0.10 (0.05-0.21) | 0.04 (0.01-0.15) |
| 3.6 Central nervous system | 0.01 (0.00-0.06) | 0.009 (0.001-0.11) | 0.01 (0.00-0.12) |
| 3.7 Liver | 0.004 (0.00-0.05) | 0.008 (0.00-0.11) | 0 (NA-0.10) |
| 3.8 Nasal and ear | 0.001 (0-0.05) | 0.003 (0.00-0.10) | 0 (NA-0.11) |
| 3.9 Respiratory system | 0.10 (0.06-0.16) | 0.107 (0.06-0.22) | 0.09 (0.05-0.21) |
| 3.10 Thymus | 0.005 (0.00-0.06) | 0.009 (0.00-0.11) | 0 (NA-0.11) |
| 3.11 Thyroid | 0.016 (0.00-0.07) | 0.009 (0.00-0.11) | 0.022 (0.00-0.13) |
| 3.12 Connective tissues | 0.004 (0.00-0.05) | 0.006 (0.00-0.10) | 0.002 (0.00-0.11) |
| 3.13 Breast | 0.022 (0.01-0.07) | 0 (NA-0.01) | 0.04 (0.01-0.15) |
| 3.14 Uterus | 0.003 (0.0001-0.011) | -- | 0.003 (0.001-0.011) |
| 3.15 Testis | 0.004 (0.00-0.10) | 0.004 (0.00-0.10) | -- |
| 3.16 Bladder | 0.003 (0.00-0.05) | 0.006 (0.00-0.10) | 0 (NA-0.11) |
| 3.17 Eye and annexa | 0.095 (0.06-0.16) | 0.102 (0.06-0.21) | 0.09 (0.04-0.21) |
| **4. MZL, NOS** | 0.05 (0.03-0.11) | 0.05 (0.02-0.15) | 0.06 (0.03-0.17) |
